# Traditional Chinese medicines alleviate experimental chronic cerebral hypoperfusion injury through multi-components and multi-target mechanisms

**DOI:** 10.3389/fphar.2025.1698436

**Published:** 2025-10-28

**Authors:** Xiao-Ting Ma, Bing-Yu Lou, Jie Liu, Bo Liu

**Affiliations:** Key Laboratory for Basic Pharmacology of Ministry of Education and Joint International Research Laboratory of Ethnomedicine, Zunyi Medical University, Zunyi, China

**Keywords:** chronic cerebral hypoperfusion, cognitive impairment, traditional Chinese medicine, multi-component, multi-target mechanisms

## Abstract

Traditional Chinese medicine (TCM) has been used in the treatment of vascular cognitive impairment and dementia caused by chronic cerebral hypoperfusion (CCH) in patients for hundreds of years. Ethnopharmacological researches have been conducted in recent years to elucidate their therapeutic effects on cognitive deficits and potential mechanisms in animal models. This manuscript critically reviewed recent 5-year experimental researches from PubMed on the topic, including 11 TCM formulae, 8 herb extracts, and 21 pure compounds extracted from TCM, including polyphenols, flavonoids, alkaloids, terpenoids, saponins, iridoid glycosides, glucosides, and others in rodent CCH models, using bilateral common carotid artery occlusion (BCCAO, 2VO), bilateral common carotid artery stenosis (BCAS), and unilateral common carotid artery occlusion (UCCAO). The underlying mechanisms are multiple, including the maintenance of blood brain barrier and endothelium integrity, the increase in cerebral blood flow, the amelioration of white matter lesions, the modulation of microglia M1/M2 phenotype, the scavenge of reactive oxidative oxygen species and reduction of proinflammatory factors, the maintenance of mitochondrial function, the inhibition of apoptosis, ferroptosis and pyroptosis, and the promotion of neuronal regeneration and angiogenesis through the regulation of gene/protein expressions, including the Toll, NF-κB, MAPK, PPARγ, and/or Nrf2 pathways. These mechanisms are not mutually exclusive, rather they play an integrated role to fortify the multi-components, multi-targets feature of TCM in the treatment of CCH and human vascular cognitive impairments.

## 1 Introduction

Chronic cerebral hypoperfusion (CCH) refers to a long-term reduction of cerebral blood flow (CBF), typically below 24–45 mL/100 g/min (normal 50–60 mL/100 g/min), and is the major cause of vascular cognitive impairments in a step-wise progression manner from mild to moderate cognitive impairment to vascular dementia (VaD) (Rajeev et al., 2023). Blood brain barrier (BBB) dysfunction occurs early in CCH, contributing to white matter damage and cognitive deficits (Xu et al., 2022). Pathologically, CCH triggers oxidative stress, neuroinflammation (Poh et al., 2022), mitochondrial dysfunction (Li et al., 2025), synaptic damage and Aβ accumulation (Wang et al., 2010), contributing to cerebral small vessel disease (CSVD), Alzheimer’s disease (AD), VaD, and other neurodegenerative diseases.

Cerebral ischemic stroke occurs not only after acute CBF reduction and focal cell death but also under CCH (Zhang et al., 2020; Zheng et al., 2023; Fadoul et al., 2024). The commonly used preclinical model for acute cerebral ischemic stroke is middle cerebral artery occlusion (MCAO) mainly characterized by cerebral infarction (Tuo et al., 2021; Zheng et al., 2023; Fadoul et al., 2024), which is different from CCH rodent models characterized mainly by cognitive impairments (Tuo et al., 2021; Kimura et al., 2025). Traditional Chinese medicines (TCMs) have been shown to be effective against both acute and chronic ischemic stroke through different mechanisms at different stages (Zhang et al., 2020; Li et al., 2022). This review focused mainly on CCH.

**FIGURE 1:**
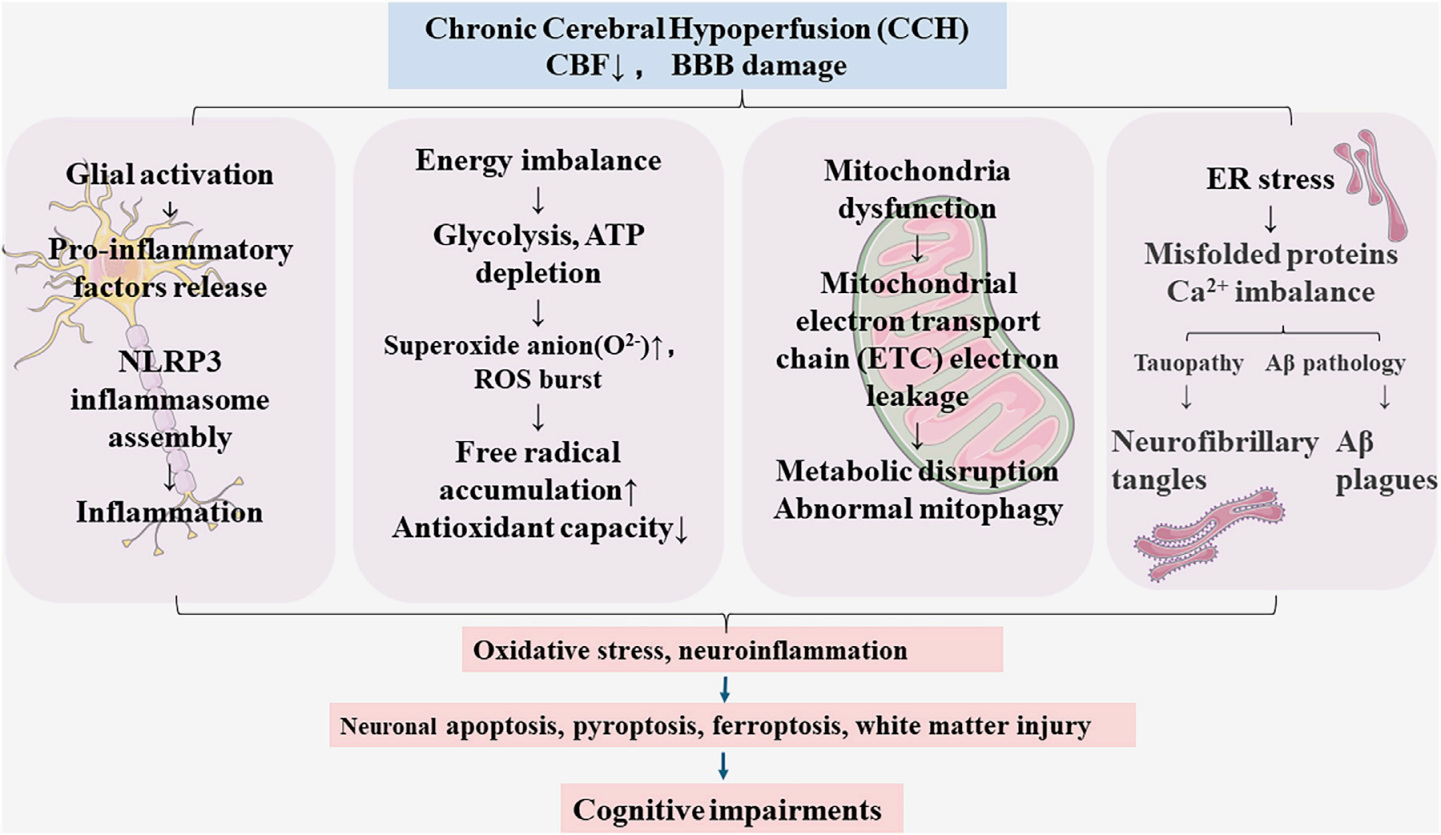
The pathological features of chronic cerebral hypoperfusion (CCH).

Figure 1 illustrates major pathophysiology of CCH: Cerebral blood flow (CBF) reduction during CCH leads to blood brain barrier dysfunction (Rajeev et al., 2022; Xu et al., 2022). The BBB tightly regulates the movements between blood and brain, BBB breakdown could cause energy imbalance, glial activation and neuroinflammation, endoplasmic reticulum (ER) stress, and mitochondrial dysfunction (Rajeev et al., 2023), which initiates a vicious cycle contributing to microglia over-activation (Zhang et al., 2024), mitochondrial dysfunction (Wang Y. et al., 2023), mitophagy dysregulation (Chen et al., 2024), oxidative damage (Rajeev et al., 2023), leading to neuronal apoptosis, ferroptosis (Zhao et al., 2022), pyroptosis (He et al., 2023), and white matter injury (Kimura et al., 2025). These pathologies ultimately result in cognitive impairments and progress to CSVD, AD, and VaD.

Traditional Chinese medicines (TCMs) have been used in the treatment of cognitive impairments, AD, VaD and various neurodegenerative diseases for thousands of years (Pei et al., 2020), and are still used today, especially for patients with mild cognitive impairment (Liang et al., 2022). The unique feature of TCM is the compound formulation, in which poly-herbs and other ingredients are composed in defined ratios to play integrated roles as “Sovereign, Minister, Assistant, and Courier” (君臣佐使 in Chinese). In 2025 Edition of Pharmacopeia of China, over 1,600 formulae are listed for treatment of various diseases and are available in Chinese drug stores (Pharmacopoeia, 2025). Front Pharmacol has published a nice review on TCM formulae against CCH (Wang Y. et al., 2023), and TCM formulae and extracts against ischemia-reperfusion induced cerebrovascular diseases (Cheng et al., 2022; Chen et al., 2024) to provide evidence of TCM as the potential research direction. The pure compounds (monomers) extracted from single herbs from TCMs, however, emerge as the novel research trend (Xie et al., 2021; Zheng et al., 2023; Zhang et al., 2024; Zhou et al., 2024).

The present manuscript aimed to review recent 5-year publications in PubMed on the protective effects of TCMs against preclinical CCH models, not to duplicate the existing reviews (Wang Y. et al., 2023). We started with 11 formulae, followed by 8 herb extracts, and finally focused on 21 pure compounds extracted from herbs against various CCH animal models, focusing on efficacy and underlying mechanisms. The bilateral common carotid artery occlusion (BCCAO, 2VO), including gradual 2VO (right artery was ligated first and the left ligation 1 week later), Unilateral common carotid artery occlusion (UCCAO), and bilateral common carotid artery stenosis (BCAS) (Tuo et al., 2021; Kimura et al., 2025) were selected, while the MCAO model was excluded as MCAO produced acute cerebral ischemic stroke compared to CCH of 2VO, UCCAO and BCAS, and had been reviewed recently (Zheng et al., 2023).

## 2 The protective effects of TCM formulae against CCH animal models

Compound formulations are the core form of clinical applications of TCM. By combining multiple medicinal herbs, formulae could achieve synergistic efficacy in the treatment of complex diseases through multi-component and multi-target mechanisms, which aligns with modern medicine’s combination therapy. The formulae strategy is also consistent with paradigm of modern systems biology and network pharmacology, offering the best therapeutic efficacy. Nine formulae against CCH models have been reviewed in Front Pharmacol (Wang Y. et al., 2023), the researches on additional 11 formulae in recent 4 years are concisely listed in Table 1, and detailed descriptions in the following text. The Chinese names of the formulae were included in the text to facilitate reader’s understanding as exampled by previous reviews on TCM formulae in Front Pharmacol (Wang Y. et al., 2022; Wang Y. et al., 2023).

**TABLE 1:** Protective effects of TCM formulae on CCH rodent models.

TCM formulae	Model	Effects	Mechanism	References
Buqi Huoxue Tong nao	2VO/Rats	Improved cognitive deficit and pathology; anti-inflammation; anti-apotosis.	↓TNFα, IL-1β, iNOS, Caspase-3; ↑AKT/PI3K; ↑LXRα/CYP7A1.	Gao et al. (2024)
Chuanzhitongluo)	BCAS/mice	Improved cognitive deficit; anti-inflammation *via* cholinergic pathway.	↑ChAT, α7nAchR; ↓NF-κB, TNFα, IL-1β, IL-6; RNA-Seq profiled targets.	Wang et al. (2024c)
Bushen-Yizhi formula	2VO/Rats (gradual)	Improved cognitive deficit and pathology; anti-oxidative stress; anti-apotosis.	↓ Mito PINK1, Parkin; ↓ LC3II/I; Cyto PINK1, Parkin; ↑ LAMP1.	(Xiao et al., 2023)
Fo-Shou-San	UCCAO/Mice	Improved cognitive deficit and pathology; anti-oxidative stress; anti-apotosis.	↓ Ferroptosis marker SLC7A11, GPX4, ROX, 4HNE; ↓ NRF2, HO-1.	Wang et al. (2023a)
Shunaoxin dropping pill	2VO/Rats (gradual)	improved cognitive deficit and hippocampal damage; anti-inflammation; improved fecal microbiota dysbiosis.	↑SOD; ↓ TNFα, IL-6, IL-10 and MDA; ↑Bacteroidetes, ↓ proteobacteria; modulated metabolomics.	Bo et al. (2023)
Modified Dioscorea pills	2VO/Rats (gradual)	Improved cognitive deficit and hippocampal damage.	↑ Expression of Ang-1, Ang-2, Tie-2, VEGF and CD43 staining.	Kuang et al. (2023)
Xinshubao tablet	BCAS/mice	Improved cognitive deficit; improve CBF; reduced WML and mitochondrial damage; anti-inflammation.	↓ NF-κB; ↓ activations of microglia and astrocyte; ↑ neurogenesis; ↑ Sox2.	Xiao et al. (2024)
Codonopsis decoction	2VO/Rats	Improved cognitive deficit; improved CBF; reduced neuron damage; anti-inflammation.	↓ CKLF1 and HIF-1α; ↓ CD16/32, TNF-α, IL-1β.	Wang et al. (2024b)
Jiawei Kongsheng Zhenzhong Pill	2VO/Rats (gradual)	Improved cognitive deficits; reduced hippocampal damage; improved synaptic plasticity of hippocampal neurons.	↑SYN, GAP43, PSD95; ↓ proBDNF/mBDNF.	Wu et al. (2025)
Taohong Siwu decoction	2VO/Rats	Improved cognitive deficits; reduced hippocampal neuron apoptosis; reduced ER stress.	↓ GRP78 (Bip), p-IRE1α, ATF6, p-eIF2α, ATF4; ↑ Bcl-2, Bax.	Fan et al. (2024)
Xi-Xian-Tong_Shuan	2VO/Rats	Improved cognitive dysfunction; reduced white matter lesions; anti-inflammation.	↑ intensity of MBP/NF200 in brain; ↓ IL-6, TNFα, MCP-1 in CSF; ↑ NeuN, MAP2 stain in the brain.	Yan et al. (2022)

### 2.1 Buqi Huoxue Tongnao prescription (BQHXTN, 补气活血通脑方)

Buqi Huoxue Tongnao prescription (BQHXTN, 补气活血通脑方) is a hospital TCM preparation derived from Erchen decoction (*Pinellia temata*, *Citrus maxima* Burm.) and *Buyang Huanwu* decoction (dried root of *Astragalus membranaceus* (Fisch.) Bunge, *Angelica sinensis*, *Paeonia lactiflora* Pall, *Ligusticum sinense*, *Prunus persica*, *Carthamus tinctorius* L.), used for treating sequelae of stroke of “Qi-deficiency” and “Blood-stasis,” and improved cerebral blood circulation and neurological functions in patients. In 2VO rats, BOHXTN (2.5–10 g/kg, 30 days) alleviated cognitive impairment, neuron loss, decreased inflammation by inhibiting IL-1β, TNF-α, cleaved caspase-3, and iNOS by activating the PI3K/AKT and LXRα/CYP7A1 signaling pathways. These findings were also observed in BV2 cells subjected to oxygen-glucose deprivation/reoxygenation (OGD/R) (Gao et al., 2024).

### 2.2 *Chuanzhitongluo capsule* (*CZTL*, *川蛭通络胶囊*)

*Chuanzhitongluo capsule* (*CZTL*, *川蛭通络胶囊*) is composed of four ingredients: Leech (Shuizhi), *Sichuan lovase* Rhizome, dried root of *Astragalus membranaceus* (Fisch.) Bunge, and dried root of *Salviae miltiorrhizae* Radix et Rhizona and is reputed for its effective protection against cerebral ischemia. In CCH mice induced by BCAS, CZTL (0.15, 0.3, 0.6, 1.2 g/kg, 30 days) improved the spatial learning and memory abilities by upregulating the choline acetyltransferase (ChAT) and α7 subunit-containing nicotinic acetylcholine receptor (α7nAChR); CZTL also inhibited the NF-κB signaling pathway and inflammatory mediators (Wang Z. et al., 2024). CZTL was also effective in improving microcirculation (Sun et al., 2022).

### 2.3 *Bushen-Yizhi formula* (*BSYZ*, *补肾益智方*)

*Bushen-Yizhi formula* (*BSYZ*, *补肾益智方*) consists of 6 herbs (*Cnidium monnieri*, (L.) Guss, *Panax ginseng* C.A.Mey, *Reynoutria multiflorum* Thunb, *Paeonia suffruticosa* Andr, *Ligustrum lucidum* Ait, and *Lycium barbarum* L.) and is used in treating cognitive impairment and neurodegenerative disorders associated with “Kidney deficiency.” In gradual 2VO rats (the left side was ligated 1 week later), BSYZ (3–6 g/kg, 4 weeks) was effective in mitigating cognitive impairments, decreasing pathological amyloid plaque formation, and alleviating oxidative stress in hippocampal regions. Furthermore, the use of OGD/R-injured PC12 cells confirmed these findings. BSYZ drug serum also increased cell survival rates while decreasing intracellular reactive oxygen species (ROS) levels (Xiao et al., 2023).

### 2.4 *Fo-Shou-San* (*FSS*, *佛手散*)

*Fo-Shou-San* (*FSS*, *佛手散*) comprises *Angelica sinensis* (Oliv.) Diels and *Ligusticum wallichii* Franch. and is used in the treatment of VaD patients. In UCCAO mice, FSS (0.5, 1, 2 g/kg, 32 days) alleviated cognitive impairments and mitigated oxidative stress by regulating the NRF2/HO-1 pathway and decreased the expression of ferroptosis markers, including SLC7A11, GPX4, ROX, and 4-hydroxynonenal (4HNE) (Wang J. et al., 2023).

### 2.5 *Shunaoxin dropping pill* (*SNX*, *舒脑心滴丸*)

*Shunaoxin dropping pill* (*SNX*, *舒脑心滴丸*) has been clinically used to treat cerebrovascular diseases. The recipe of SNX is composed of two herbs, namely, Chuanxiong (*Ligusticum chuanxiong* Hort) and Danggui (*Angelica sinensis* (Oliv.) Diels). SNX (60 and 200 mg/kg, 28 days) alleviated cognitive deficits and restored imbalances in fecal microbiota and serum metabolites in rats with CCH. SNX failed to prevent cognitive impairment in CCH rats treated with antibiotics, indicating the importance of the microbiota-gut-brain axis as a potential therapeutic target for treating cognitive impairment induced by CCH (Bo et al., 2023).

### 2.6 *Modified Dioscorea pills* (*MDP*, *改良薯蓣丸*)

*Modified Dioscorea pills* (*MDP*, *改良薯蓣丸*) are composed of 14 herbal ingredients: *Dioscorea rhizome*, *Rehmannia glutinosa*, *Polygonum multiflorum*, *Angelica sinensis*, *Eucommia ulmoides*, *L. chuanxiong*, *Codonopsis pilosula*, *Poria cocos*, *Atractylodes macrocephala*, *Paeonia lactiflora*, *L. barbarum*, *Acorus tatarinowii*, *Polygala tenuifolia*, and *Schisandra chinensis*. MDP (1 mL/100 g, 45 days) mitigated neuronal loss and facilitated the repair of impaired hippocampal structures in CCH rats. MDP also enhanced angiogenesis and remodeled microcirculation in CCH rats through the Ang/Tie signaling pathway (Kuang et al., 2023).

### 2.7 *Xinshubao tablet* (*XSB*, *心舒宝片*)

*Xinshubao tablet* (*XSB*, *心舒宝片*) is a patented TCM formula that comprises five Chinese herbal medicines, including air-dried root of *Salvia miltiorrhiza* (Danshen), *P. lactiflora Pall* (Baishao), *Acanthopanax senticosus* (Ciwujia), air-dried root of *Curcuma longal* L. (Yujin), and air-dried mature fructus of *Crataegus pinnatifida* (Shanzha). XSB is used for cardiovascular diseases and dementia in patients. In BCAS mice, XSB (3.75–15 g/kg, 8 weeks) improved cognitive deficits and brain pathology through multiple mechanisms: improving cerebral blood flow (CBF), reducing white matter lesions, inhibiting glial cell activation, attenuating neuroinflammation and promoting neurogenesis. Importantly, the NF-κB signaling pathway was identified as a central component in mediating these protective effects (Xiao et al., 2024).

### 2.8 *Codonopsis decoction* (*党参汤*)

*Codonopsis decoction*(*党参汤*) is mainly composed of *C. pilosula* (Franch.) Nannf. and dried fruits of *Ginkgo Biloba* L. extract and is known for “invigorating the spleen,” “nourishing the lungs,” “promoting blood circulation,” and “generating fluid” properties. *Codonopsis* decoction (2.7–10.8 g/kg, 4 weeks) demonstrated protective effects against CCH rats by effectively reducing brain damage, improving CBF in ischemic regions, and enhancing learning and memory capabilities, possibly through the reduction of chemokines, hypoxia-inducible factors, and neuroinflammatory mediators (Wang J. et al., 2024).

### 2.9 *Jiawei Kongsheng Zhenzhong Pill* (*JKZP*, *加味孔圣枕中丹*)

*Jiawei Kongsheng Zhenzhong Pill* (*JKZP*, *加味孔圣枕中丹*) is derived from the “Kongsheng Zhenzhong Pill” in “Thousand-Golden-Prescriptions,” and *S. miltiorrhiza* Bunge, *Conioselinum anthriscoides* (H.Boissieu), *Cornus officinalis* Sieb et Zucc., and *Cistanche deserticola* Ma. were added to strengthen the efficacy in “Tonifying the kidneys,” benefiting the “Vital essence,” activating blood circulation, and removing blood stasis. In gradual 2VO rats, JKZP (11.3 g/kg, 60 days) treatment for 60 days improved learning and memory ability, alleviated neuronal and synaptic structural damage in the hippocampal CA1 region. It reversed the reduction in dendritic spine density and increased the expression of synaptic-related proteins, such as synaptophysin (SYN), growth-associated protein 43 (GAP43), and postsynaptic density protein 95 (PSD95). Additionally, JKZP significantly reduced the ratio of pro-brain-derived neurotrophic factor (pro-BDNF) to mature brain-derived neurotrophic factor (mBDNF) by activating S100A10/tPA, thereby improving synaptic plasticity in hippocampal neurons. Using primary hippocampal neuron cultures under OGD/R, the efficacy of JKZP drug serum and the importance of the S100A10/tPA/BDNF signaling *via* sh-S100A10 were verified (Wu et al., 2025).

### 2.10 *Taohong Siwu decoction* (*TSD*, *桃红四物汤*)

*Taohong Siwu decoction* (*TSD*, *桃红四物汤*) is a classic TCM formula composed of *R. glutinosa* (Gaertn.) DC., *Juglans regia* L., *Angelica sinensis* (Oliv.) Diels), *Carthamus tinctorius* L., *P. lactiflora* Pall, *C. anthriscoides* (H. Boissieu), and is used for the treatment of vascular diseases, including VaD in clinics. After 2VO surgery in rats, TSD (4.5–13.5 g/kg, 4 weeks) improved cognitive deficits and ameliorated neuron damage in the CA1 region of hippocampus. Mechanistically, TSD attenuated endoplasmic reticulum stress (ERs) and the unfolded protein response (UPR) responses, and reduced apoptosis (Fan et al., 2024).

### 2.11 *Xi-Xian-Tong-Shuan* (*豨莶通栓胶囊*)

*Xi-Xian-Tong-Shuan* (*豨莶通栓胶囊*) is composed of Herba Siegesbeckiae (broiled in honey wine) and 13 other ingredients that could remove “Wind-phlegm,” produce “Tendon relaxation,” cause “Meridian activation,” activate blood circulation by removal of stasis, and produce “consciousness-restoring” effects, and is mainly used for hemiplegia, hemianesthesia, skewed mouth, deviated tongue, and slurred speech. In 2VO rats, this formula (500 mg/kg, 42 days) improved cognitive impairment in Morris water maze, ameliorated white matter lesions and reduced neuron loss in the cortex and hippocampus, decreased the release of IL-6, TNF-α, MCP-1, and IL-33 in the cerebrospinal fluid and in plasma (Yan et al., 2022).

Front Pharmacol has already published reviews on TCM formulae against 2VO CCH rodent models include Phlegm-purging decoction (涤痰汤), Shenmayizhi decoction (参麻益智汤), Zuogui Pill (左归丸), Kai Xin San (开心散), Qufeng Tongqiao decoction (祛风通窍汤), Fuzhi capsule (复智胶囊), Modified decoction of Rehmanniae (地黄饮子加减方), Naomaitai capsule (脑脉泰胶囊) and Shenzhi Jiannao Formula (参知健脑方) (Wang Y. et al., 2023). In addition, Danggui Shaoyao San (当归勺药散) (Cheng et al., 2022) and Taohong Siwu decoction (桃红四物汤) have been reported to be effective against MCAO model (Chen et al., 2024). In general, TCM formulae are more effective than single herb, extracts, or pure compounds, as multi-components function in an integrated manner to enhance pharmacological efficacy and reduce toxicity.

## 3 The protective effects of TCM extracts against CCH animal models

In addition to TCM formulae, a lot of studies are conducted on total extracts from herbs including water extracts, ethanol extracts, total flavonoids, total alkaloids, *etc.*, Table 2 summarized 8 TCM extracts against CCH in animal models.

**TABLE 2:** Protective effects of TCM herb extracts on chronic cerebral hypoperfusion.

TCM extracts	Model	Effects	Mechanism	References
Epimedium flavonoids	2VO/Rats	Improved cognitive deficits; preserved myelin and synapse ultrastructure; reduced neuron loss.	↑BDNF, NRG1, PI3K; ↓Lingo-1, Fyn, ROCK2; ↑PSD95, Synaptic plasticity-related proteins.	Niu et al. (2020a), Niu et al. (2020b)
*Scutellaria baicalensis* extracts	2VO/Rats	Improved cognitive deficits; anti-inflammation.	↓p-ERK, p-JNK, p-P38; ↓Activation of macroglia.	Hwang et al. (2011)
Fructus mume extracts	2VO/Rats	Improved cognitive deficits; anti-neuronal apoptosis; anti-inflammation.	↓Death of neurons; ↓p38 MAPK phosphorylation; ↓COX-2, IL-1β, IL-6.	Lee et al. (2015)
*Aster ageratoides *extract	2VO/Rats	Improved cognitive deficits; improves the deterioration of the hippocampal structure.	↓ Neuronal loss; ↓ Microglial activation.	Jeong et al. (2020)
Radix Polygoni Multiflori (Raw and Processed)	2VO/Rats	Improved cognitive deficits; reduced neuron loss in the CA1 region of the hippocampus.	Both RPM and PPM ameliorated 2VO-related metabolomic abnormalities.	Wu et al. (2022)
*Nigella sativa* extract and active ingredient thymoquinone	2VO/Rats	Improved cognitive deficits.	↑SOD; ↓MDA, AChE activity.	Fanoudi et al. (2019)
*Ginkgo biloba* extracts (GBE)	2VO/Rats	Suppressed activation of microglia and astrocytes.	↓TLR4, TNF-α, IL-1β, IL-6; ↓MyD88, RACE, Ang-II; ↓ERK, p38, JNK; ↑ChAT+ neurons.	Kim et al. (2016)
Polysaccharide from *Ganoderma lucidum*	2VO/Mice	Improved cognitive deficits, regulated Treg cells.	↑Foxp3 (+) Treg cell; ↑IL-10, TGF-β1.	Zhang et al. (2022)

### 3.1 Epimedium flavonoids (EF)

*Epimedium flavonoids* (EF) were mainly extracted from *Epimedium brevicornum* Maxim., EF 50–200 mg/kg, 12 weeks improved cognitive impairment, reduced white matter lesions and maintained ultrastructure of myelin sheaths and neurons in 2VO rats. The loss of oligodendrocytes was prevented. Mechanistically, EF inhibited the Lingo-1/Fyn/ROCK pathways and activated the BDNF/NRG1/PI3K pathways (Niu et al., 2020b). EF also reduced 2VO-induced apoptosis and neuron loss and ameliorated synapse ultrastructure damage. EF protected synaptic plasticity by increasing the expression of synaptophysin, synaptotagmin-I, synapsin I, PSD-95, p-NNMDAR2B and p-CaMKIIα; and protected neuronal dendrites *via* increasing MAP2 and NF200 protein expression in the hippocampus of 2VO rats. The NRG1/ErbB4 and BDNF/Fyn signaling pathways were involved in EF protection (Niu et al., 2020a).

### 3.2 *Scutellaria baicalensis* (Lamiaceae)

*Scutellaria baicalensis* (Lamiaceae) *water extracts* contain several flavonoids, such as wogonin, baicalin, and oroxylin and were orally administered (100 and 200 mg/kg/d) to 2VO rats (starting 20 days after 2VO for 40 days) and in LPS-infused rats *via* minipump (staring 7 days after minipump implant for 32 days). *Scutellaria baicalensis* extracts improved cognitive deficit in CCH rats and in chronic LPS-infusion rats. The mechanism is probably mediated through hippocampal mitogen-activated protein kinases (pERK, pJNK, and p-p38) signaling and reduced microglial activation (OX-6 stain) (Hwang et al., 2011).

### 3.3 *Fructus mume* (Sieb.)

*Fructus mume* (Sieb.) *ethanol extracts* (200 mg/kg/d, po, initiated 21 days after 2VO surgery and continued for 42 days) demonstrated neuroprotective effects in CCH rats by preserving myelin basic protein expression in hippocampal and white matter regions and by decreasing neuroinflammatory markers including COX-2, IL-1β, and IL-6 in the hippocampus, while concurrently inhibiting the activation of TLR4/MyD88 and modulating the p38 mitogen-activated protein kinase (MAPK) signaling pathways (Lee et al., 2015).

### 3.4 *Aster ageratoides* Turcz

*Aster ageratoides* Turcz *water extracts* (AAE, 10, 50 mg/kg, ip, for 14 days) improved cognitive deficits in 2VO rats and in Scopolamine-treated rats. 2VO-induced neuron loss and macroglia activation in the CA1 region of hippocampus were ameliorated following AAE treatments (Jeong et al., 2020).

### 3.5 Dried-root of *Polygonum multiflorum*

Dried-root of *Polygonum multiflorum* is usually processed for the use in TCM. Raw Polygoni Multiflori (RPM) was compared to Processed Polygoni Multiflori (PPM) for their efficacy in the treatment of 2VO rats. Four days after 2VO, the surviving rats were given RPM or PPM (2.0 g/kg, po for 28 days). Both RPM and PPM were effective in improving cognitive deficits, ameliorated neuron loss in the CA1 region of hippocampus. In metabolomic studies, both RPM and PPM ameliorated 2VO-induced abnormal vitamin B6 metabolism, pentose phosphate pathways, taurine and hypotaurine metabolism. The metabolism of cysteine and methionine was regulated only by RPM, and riboflavin metabolism was modulated only by PPM. The results suggested that raw and processed PM had comparable efficacy in the treatment of dementia with some mechanistic differences (Wu et al., 2022).

### 3.6 Nigella sativa seed hydroalcoholic extracts

*Nigella sativa seed hydroalcoholic extracts* (NSE, 100, 200, 400 mg/kg, ip for 10 days) improved cognitive deficits in 2VO rats. NSE ameliorated 2VO-elevated lipid peroxidation (MDA) levels, while increased SOD activity in the hippocampus. The 2VO-increased AChE activity was also reduced by NSE. Its active ingredient thymoquinone (TQ 10, 20, 40 mg/kg, ip) also produced similar effects in a dose-dependent manner (Fanoudi et al., 2019).

### 3.7 *Ginkgo biloba L.* extracts

*Ginkgo biloba* L. *extracts* (GBE 5, 10, 20, 40 mg/kg, po) administration to 2VO rats staring 21 days after 2VO surgery for 42 days suppressed the activation of microglia and astrocytes in the brain, reduced proinflammatory cytokines (TNF-α, IL-1β, and IL-6), TLR4, MyD88, RAGE, Ang-II, and phosphorylated MAPKs (ERK, p38, and JNK). GBE treatment restored the ChAT-positive cholinergic neurons in the basal forebrain (Kim et al., 2016).

### 3.8 Polysaccharide

*Polysaccharide from Ganoderma lucidum* (Lingzhi) (30 mg/kg, po, 30 days) alleviated cognitive impairment in gradual 2VO mice. Flow cytometry found the treatment of Polysaccharide from *G. lucidum* increased CD4(+)CD25(+)Foxp3(+) regulatory T cells, and subsequently increased levels of IL-10 and TGF-β1, to ameliorate abnormal metabolism (Zhang et al., 2022).

## 4 The protective effects of TCM pure compounds against CCH animal models

**FIGURE 2:**
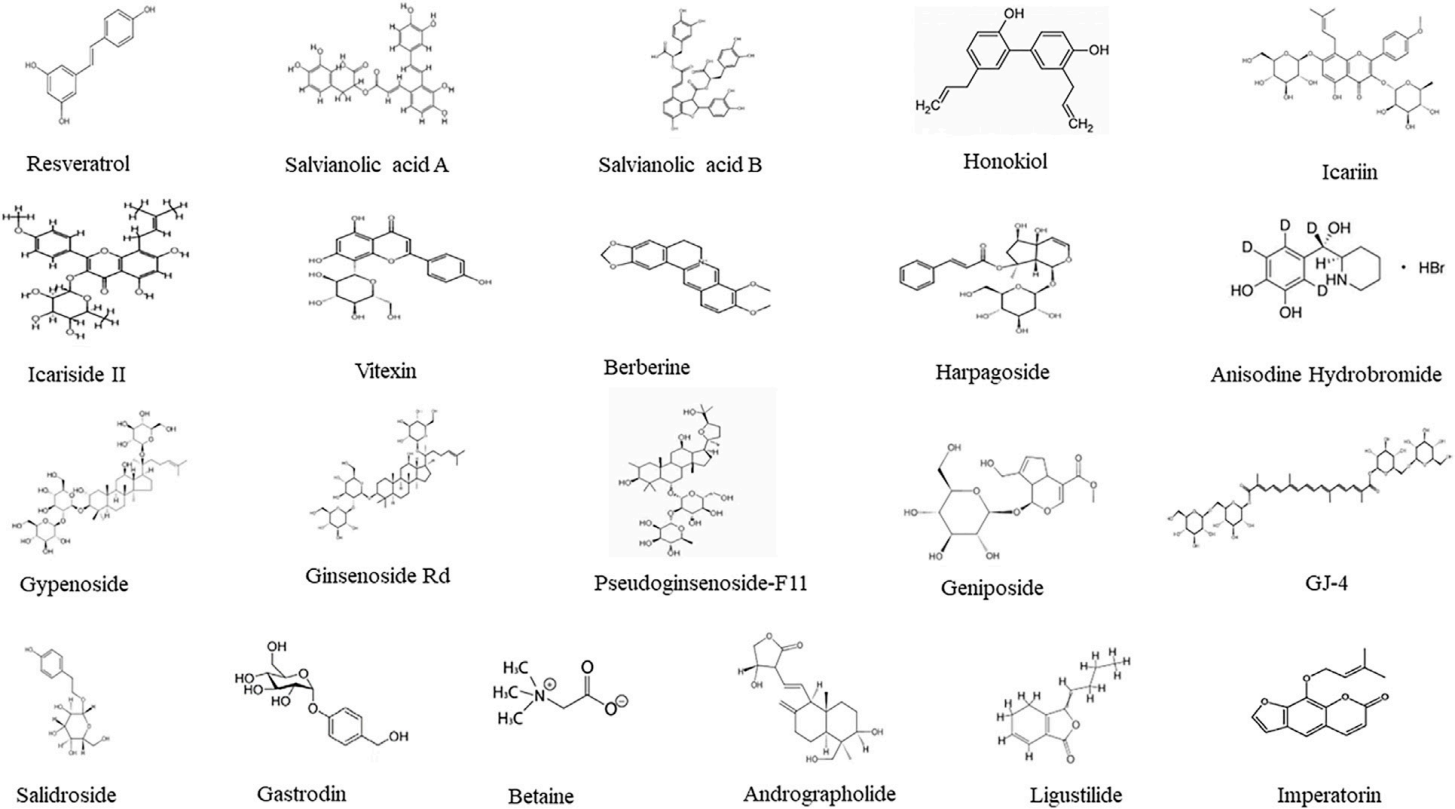
Structures of 21 TCM pure compounds effective against CCH.

Further research utilized purified monomeric component from TCM that have clearer structural definitions and controllable purity levels, producing reproducible results compared to TCM formulae and extracts (Xie et al., 2021). It should be mentioned that these pure compounds existed in many medicinal herbs although only the initial isolated herbs were mentioned, and the pure compounds helped to elucidate the mechanisms of protection. Table 3 summarized 21 of TCM monomers against CCH in animal models. The structures of these 21 TCM monomers are presented in Figure 2.

**TABLE 3:** Protective effects of TCM pure compounds on chronic cerebral hypoperfusion.

Classification	Monomer	Model	Effects	Mechanism	References
Polyphenol	Resveratrol	BCAS/Mice	Improved cognitive deficits without affecting CBF; reduced cholinergic cell loss.	↑ChAT neurons.	Fagerli et al. (2024)
	Salvianolic acid A	2VO/Rats	Improved cognitive deficits and pathology; suppressed neuronal apoptosis; anti-inflammation.	↓Caspase-3; ↓TNF-α, IL-1β, and IL-6; ↓NF-κB; ↑Cryab, Drd2.	Yang et al. (2022)
	Salvianolic acid B	2VO/Rats	Improved cognitive deficits; suppressed neuronal apoptosis.	↑IGF-1; ↑p-Akt.	Ma et al. (2017)
	Honokiol	BCAS/Mice	Improved cognitive deficits; reduced myelin injury; promoted oligodendrocyte regeneration.	↑Akt/p-Akt, mTOR/p-mTOR; ↑MBP, Sox10, MAG; ↓NG2.	Zhang et al. (2023)
Flavonoids	Icariin	2VO/Rats	Improved cognitive deficits; reduced hippocampal damage and Aβ deposition.	↑ADAM10, IDE; ↓Aβ, APP, BACE1, TGF-β1, Smad2/3.	Li et al. (2015)
	Icarrin	2VO/Rats	Improved cognitive deficits; ameliorated oxidative stress.	↑SOD; ↓MDA; ↑ACh, AChE.	Xu et al. (2009)
	Icariside II	2VO/Rats	Improved cognitive deficits; reduced hippocampal neuron loss; reduced Aβ accumulation.	↓Aβ, APP, BACE1; ↑ADAM10, IDE; ↑BDNF, TrkB, p-Akt, p-CREB; PPARγ.	Yin et al. (2018)
	Icariside II	2VO/Rats	Improved cognitive deficits and hippocampal damage; promoted neuronal axon regeneration.	↑GAP43, MAP2; ↓Nogo-A.	Liu et al. (2019)
Alkaloids	Vitexin	2VO/Rats	Improved cognitive deficits; suppressed neuronal apoptosis; anti-inflammation.	↑Epac1, Epac2, Rap1, p-ERK; ↓NLRP3, caspase-1, IL-1β, IL-6, caspase-3.	Zhang et al. (2021)
	Berberine	2VO/Rats	Improved cognitive deficits; reduced hippocampal damage and apoptosis.	↓Apoptotic cell death, Caspase 3, MDA; ↑ SOD, CAT.	Aski et al. (2018), Pirmoradi et al. (2019)
	Berberine	2VO/Rats	Improved cognitive deficits and pathology; reduced neuron loss.	↑ Hippocampal p-ERK, Nrf2; ↓VEGF-A, MMP-9.	Wang et al. (2022a)
	Betaine	2VO/Rats	Improved cognitive deficits; anti-oxidative stress.	↑PSD93, PSD95, MAP2; ↑SOD, GSH; ↓ROS, MDA.	Nie et al. (2016)
	Anisodine Hydrobromide	2VO/Rats	Improved cognitive deficits; reduced neuron loss; modulated cholinergic system.	↑ Brain 5-HT; ↓AchE; ↑ Bcl2, p-Akt, GSK-3β; ↓Bax.	Chen et al. (2017)
Glucosides	Salidroside	2VO/Rats	Improved cognitive deficits; reduced neuron loss and apoptosis; ameliorated LTP inhibition	↓ Bax/Bcl-2 ratio and caspase-3 activation.	Yan et al. (2015)
	Salidroside	BCAS/Mice	Improved cognitive deficits; reduced neuroinflammation.	↓ Cleaved casoase 3; ↓ iNOS, TNF-α, and IL-1β by shifting microglia M1 to M2.	Ji et al. (2025)
	Salidroside	BCAS/Rats	Improved cognitive deficits; protected the BBB; promoted angiogenesis.	↑Notch1, Hes1, Hes5, and ITGB1.	Zhilan et al. (2025)
	Gastrodin	2VO/Rats	Improved cognitive deficits and neuron loss; improved energy metabolism disorders.	↓ Aβ and Tau Protein Expression; ↓ Mitochondrial dysfunction in HT-22 cells.	Wu et al. (2023)
Iridoid glycoside	Harpagoside	2VO/Rats	Improved cognitive deficits; reduced hippocampal neuron loss.	↑ p-PTEN, p-Akt; ↓ GSK-3β.	Chen et al. (2018)
	Geniposide	2VO/Rats	Improved cognitive deficits and pathology; anti-inflammation.	↓ GFAP, iNOS, NF-κB; ↓ TNF-α and IL-6.	Li et al. (2020)
	GJ-4,a Geniposide derivative	BCAS/Mice	Improved cognitive deficits and CBF; reduced WMI; improved lipid metabolism; anti-oxidative stress	↑ Bcl-2/Bax ratio; ↓ Caspase-3; ↓ ROS; ↑SOD, GSH, Nrf2/HO-1.	Pang et al. (2024)
Diterpeniod	Andrographolide	2VO/Rats	Improved cognitive deficits and pathology; anti-apotosis.	↓TNF-α, IL-1β, caspase-3; ↓GFAP; ↑BDNF, TrkB; ↓ p-PETN and ↑ p-Akt.	Wang et al. (2019), Wang et al. (2020)
Trierpenoid saponins	Gypenoside	2VO/rats	Improved cognitive deficits; attenuated white matter lesions; anti-oxidative stress; anti-inflammation.	↓ Kluver-Barrera staining; ↓ 4-HNE, 8-OHdG, and GAFP positive cells; ↑SOD; ↓MDA.	Zhang et al. (2011)
	Ginsenoside Rd	BCAS/Mice	Improved cognitive deficits; promoted neuronal survival; decreased apoptosis.	↑ BDNF; ↑ p300/CBP; ↓ HDAC2; ↓ Casp-3, Ac-H3, HDAC2 in OGD/R neurons.	Wan et al. (2017)
	Pseudoginsenoside-F11	2VO/Rats (Gradual)	Improved cognitive deficits; reduced white matter injury; modulated mTOR-mediated autophagy.	↓ Axon demyelination and loss; ↓ p-mTOR, p-ULK1; modulated autophagy proteins.	Wang et al. (2024a)
Dihydrophthalide	Ligustilide	2VO/Rats	Improved cognitive deficit; prevented neuron loss and apoptosis; reduced dendritic damage.	↑ Nissl+, NeuN+ neurons; ↓ Caspase-3, GFAP; MAP2.	Feng et al. (2012)
	Ligustilide	2VO/Rats	Improved cognitive deficits; reduced ER stress and oxidative stress.	↑ Nissl+ neurons; ↑ SOD, CAT, GSH-Px; ↓ MDA, Bip, p-IRE1α, XBP1, CHOP, ↑ SIRT1.	Peng et al. (2022)
Furanocoumarin	Imperatorin	2VO/Rats	Improved cognitive deficits; attenuated neuronal damage; improved synaptic ultrastructure.	↑ Bcl-2; Bax, Caspase-3; ↑ PSD-95.	Huang et al. (2021)

### 4.1 The neuroprotective effects of polyphenol compounds

#### 4.1.1 *Resveratrol* (RSV)

*Resveratrol* (RSV) is a natural polyphenol found in grape and many herbs RSV (10 mg/kg, ip, twice/week for 10 injections) alleviated cognitive deficits in a gradual BCAS mouse model, evidenced by novel object recognition test. This protection was not due to increases in CBF as determined by Laser Speckle Contrast Image, but was associated with the preservation of cholinergic neurons in the septal nucleus, offering potential therapeutic insights for neurodegenerative diseases beyond AD, including vascular cognitive impairment and dementia (VCID) (Fagerli et al., 2024).

#### 4.1.2 Salvianolic acid A (SalA) and Salvianolic acid B (SalB)

*Salvianolic acid A* (SalA) and *Salvianolic acid B* (SalB) are from *Salvia miltiorrhiza* Bunge, a traditional Chinese medicinal herb widely used for cardiovascular and cerebrovascular diseases. SalA (5, 10, 20 mg/kg, po for 28 days) could improve the cognitive function of 2VO rats, reduce pathological damage of cortex and hippocampus, inhibit neuroinflammation and apoptosis, and suppress the activation of NF-κB by regulating the Drd2/Cryab pathway. These findings were further verified in SH-SY5Y cells injured by hypoglycemia and hypoxia (Yang et al., 2022). SalB (20 mg/kg, po for 6 weeks) ameliorated cognitive deficits in 2VO rats, and reduced neuron damage and apoptosis in the CA1 region of hippocampus. The protection is probably mediated through recover of hippocampal IGF-1/Akt pathway. Although CCH did not alter hippocampal Akt levels, the p-Akt was decreased along with decreased IGF-1, which was returned to normal after SalB treatment at both mRNA and protein levels (Ma et al., 2017).

#### 4.1.3 Honokiol and magnolol

Honokiol and magnolol are two major compounds derived from *Magnolia officinalis* L., both are shown to facilitate the differentiation of primary oligodendrocyte precursor cells (OPCs) into mature oligodendrocytes. Honokiol (10 mg/kg, ip for 30 days) improved cognitive deficits in BACS mice, ameliorated myelin injury in several brain regions *via* Black-Gold staining and electron microscopy. Although Honokiol did not affect CBF, it did inhibit activation of astrocytes (GFAP) but not microglia (Iba1). Honokiol could increase p-Akt and p-mTOR in BACS mice and in OPC cell cultures, suggesting its beneficial effects could be associated with OPC differentiation (Zhang et al., 2023).

### 4.2 The neuroprotective effects of flavonoid compounds

#### 4.2.1 Icariin (ICA)

*Icariin* (*ICA*) is a flavonoid compound and the primary active ingredient of plants of *Epimedium brevicornu* Maxim. ICA (10–120 mg/kg, po for 1–3 months) positively modulated multiple targets associated with Aβ pathways and thus, may be beneficial in attenuating the level of Aβ in the VaD or AD brain by decreasing the production of Aβ (*via* downregulation of beta-site APP cleaving enzyme 1 (BACE1) and upregulation of ADAM10) and by increasing the degradation of Aβ (upregulation of IDE). Furthermore, suppression of TGF-β1 signaling pathway may also be involved in the ICA-induced reduction of Aβ (Li et al., 2015). ICA could also alleviate oxidative stress in the brain caused by 2VO, as evidenced by a reduction in MDA levels and the preservation of superoxide dismutase (SOD) activity. Furthermore, ICA prevented the decline in hippocampal levels of acetylcholine, acetylcholinesterase, and choline acetyltransferase associated with CCH (Xu et al., 2009).

#### 4.2.2 Icariside II (ICS II)

*Icariside II* (*ICS II*) is the main metabolites of ICA. ICS II (4–16 mg/kg, po for 28 days) improved spatial learning and memory in 2VO rats in MWM tests, and ameliorated hippocampal damage (HE staining) and neuron loss (Nissl staining). Aβ accumulation was reduced dose-dependently, probably by downregulating amyloid precursor protein (APP) and β-secretase 1 (BACE1), as well as by upregulating ADAM10 and IDE. The CCH-suppressed BDNF/TrkB, p-Akt/Akt, and p-CREB pathways were recovered by ICS II, and CCH-inhibited PPAR-α and PPAR-γ were returned to Sham levels (Yin et al., 2018). ICS II (4 and 8 mg/kg/day, po for 4, 8 and 12 weeks) reduced the escape latency and increased the time in target quadrant in MWM, with the high dose more effective. Hippocampal lesions were ameliorated. Notably, ICS II promoted neuron axon regeneration and repair by increasing GAP-43 and MAP-2 expression and reducing Nogo-A expression in the CA1 of the hippocampus *via* immunohistochemical staining (Liu et al., 2019).

#### 4.2.3 Vitexin

*Vitexin* is a naturally occurring flavonoid glycoside extracted from *Crataegus pinnatifida* Bunge, *Vigna radiata*, *Passiflora incarnata* and other herbs, and is known for its antioxidant and anti-inflammatory properties. Vitexin (2.5, 5, 10 mg/kg, ip for 4 weeks) improved cognitive decline in 2VO rats, reduced pathological damage in the cortex and hippocampus. Vitexin mitigated inflammation-induced damage in CCH by downregulating the expression of NLRP3, caspase-1, IL-1β, IL-6, and cleaved caspase-3. The CCH-decreased levels of exchange protein directly activated by cAMP 1 (Epac1), Epac2, Ras-associated protein 1 (Rap1), and p-ERK1/2 were reversed by Vitexin. These *in vivo* findings were further confirmed in hippocampal HT22 cells under OGD/R (Zhang et al., 2021).

### 4.3 The neuroprotective effects of alkaloids

#### 4.3.1 Berberine (BBR)

Berberine (BBR) is a benzylisoquinoline alkaloid extracted from many herbs such as Berberis, *Coptis chinensis*, *B*. *integerrima* or *B. vulgaris* with a long history of medicinal applications in traditional medicines with many pharmacological effects. BBR (50–100 mg/kg, po for up to 2 months) ameliorated 2VO-induced cognitive impairment and neuronal damages in the CA1 hippocampal subregion and in frontal cortex. 2VO-induced hippocampal neuronal loss and apoptosis were alleviated by BBR, probably mediated by reducing MDA, increasing antioxidant SOD and CAT activities, as well as by decreasing caspase-3 to exert anti-apoptotic effects (Aski et al., 2018; Pirmoradi et al., 2019). In 2VO-induced CCH rats, BBR (90 mg/kg, po for 42 days) reduced escape latency and increased time crossing platform in MWM test. The fluorescence intensity of NeuN in the cortex, hippocampus CA1 and CA3 was increased compared to CCH rats. Notably, the expression of Nrf2 and p-ERK proteins in the brain was increased as evidenced by immunofluorescences stain and WB. BBR could also reduce BBB injury by inhibiting CCH-elevated VEGFA and MMP9, while increasing SMA and Collogen IV with immunofluorescence stain (Wang N. et al., 2022).

#### 4.3.2 Betaine

*Betaine* is a kind of water-soluble quaternary amine-type alkaloid widely existing in food, such as wheat germ, beet, spinach, shrimp and wolfberry. Betaine (12.5 mg/kg/d *via* drinking water for 14 days) decreased the escape latency and increased the time spent in the target quadrant of 2VO rats, indicating the improvement of memory function. Betaine also improved synaptic function by upregulating postsynaptic proteins (PSD93, PSD95, and MAP2). At the same time, Betaine could also upregulate SOD and GSH, and downregulate ROS and MDA to normal levels, thus playing an antioxidative stress role (Nie et al., 2016).

#### 4.3.3 Anisodine Hydrobromide (AH)

*Anisodine Hydrobromide* (AH) is a tropane alkaloid drug derived from the plant *Anisodus tanguticus* Maxim. It is used in the treatment of various vascular and neurological conditions. In CCH rats, low dose of AH (0.3–1.2 mg/kg) improved cognitive impairments in MWM test. TUNEL and Nissl staining showed amelioration of CCH-induced apoptosis and neuron loss. The cholinergic system was modulated with AChE activity reduction, while increasing 5-HT. Protein expression of Bcl2, p-Akt and p-GSK-3β increased, while Bax decreased, consistent with reduced apoptosis (Chen et al., 2017).

### 4.4 The neuroprotective effects of glucosides

#### 4.4.1 Salidroside

*Salidroside* is primarily derived from plants of the Rhodiola genus (such as *Rhodiola rosea* and *Rhodiola crenulata*). Salidroside (20 mg/kg, po, 35 days) improved cognitive deficits in 2VO rats and attenuated apoptosis in the CA1 region of rat hippocampus. CCH-impaired long-term potentiation (LTP) was ameliorated by Salidroside, and CCH-activated caspase-3 and elevated Bax/Bcl2 ratio were also inhibited by Salidroside (Yan et al., 2015). In BCAS-induced CCH mouse models, BCAS produced a significant decrease in CBF, leading to cognitive impairment and neuronal apoptosis. Salidroside (10 mg/kg, ip, for 35 days) improved cognitive deficit in MWM test, despite its effects on CBF and Nissl staining were not mentioned, it shifted microglial polarization from the pro-inflammatory M1 phenotype to the anti-inflammatory M2 phenotype, thus suppressing pro-inflammatory cytokine release, and improving neuronal survival (Ji et al., 2025). Salidroside was also found to alleviate blood-brain barrier (BBB) disruption induced by cerebral hypoperfusion and enhance angiogenesis by promoting endothelial cell budding. Further studies revealed that Salidroside upregulated Notch1, Hes1, Hes5, and ITGB1 to activate the Notch signaling pathway, which in turn promotes angiogenesis and protects the BBB integrity (Zhilan et al., 2025).

#### 4.4.2 Gastrodin

*Gastrodin* is a glucoside of Gastrodigenin, both are derived from *Gastrodia elata* Bl, a traditional Chinese herbal medicine used for centuries to treat cognitive impairment, ischemic stroke, epilepsy, and dizziness. Gastrodin and Gastrodigenin (25 and 50 mg/kg, po for 4 weeks) improved the cognitive impairment of 2VO rats by reducing the expression of Aβ and inhibiting the phosphorylation of Tau protein. Brain metabolomics revealed that CCH-disrupted energy metabolism such as glycolysis, TCA cycle and pentose phosphate pathways were improved by Gastrodin and Gastrodigenin. Both compounds also protected HT-22 hippocampal cells from hydrogen peroxide (H_2_O_2_)-induced damage by improving the mitochondrial function (Wu et al., 2023).

### 4.5 The neuroprotective effects of iridoid glycoside

#### 4.5.1 Harpagoside

*Harpagoside* is a bioactive iridoid glycoside primarily extracted from the root of Devil’s Claw (*Harpagophytum procumbens* (Burch.) DC), a medicinal plant native to Southern Africa and traditionally used for its anti-inflammatory and analgesic properties. Harpagoside (15 mg/kg, po for 2 months) improved cognitive deficit in MWM and avoidance tests and reduced neuron loss as evidenced in Nissl staining. Harpagoside increased p-PTEN and p-Akt expression, enhanced Akt activity and suppress GSK-3β activity, both of which are downstream effectors of PTEN (Chen et al., 2018).

#### 4.5.2 Geniposide

*Geniposide* is a bioactive iridoid glycoside primarily extracted from the fruit of *Gardenia jasminoides* Ellis (Cape jasmine or Zhi zi), a traditional Chinese medicinal plant known for its anti-inflammatory and neuroprotective effects. Geniposide (50, 100 mg/kg, po, 4 weeks) effectively mitigated cognitive decline caused by CCH in rats. It likely alleviated neuroinflammation during chronic cerebral ischemia by downregulating iNOS and NF-κB expression and inhibiting the release of inflammatory factors such as TNF-α and IL-6 (Li et al., 2020).

#### 4.5.3 GJ-4, a geniposide derivative

GJ-4, a geniposide derivative, also alleviated cognitive impairments in BCAS mice at the dose of 50 mg/kg, po, for 28 days. GJ-4 improved CBF and reduced the white matter lesions. GJ-4 reduced oxidative stress and neuron apoptosis by decreasing ROS levels and caspase-3, and increasing the Nrf2/HO-1 antioxidant pathway. The Bcl-2/Bax ratio and antioxidant SOD and GSH were also increased (Pang et al., 2024).

### 4.6 The neuroprotective effects of terpenoids and triterpenoid saponins

#### 4.6.1 Andrographolide

Andrographolide is a bioactive diterpenoid lactone primarily extracted from the leaves and stems of *Andrographis paniculata* Burm.f. (commonly known as “green chiretta” or “king of bitters”), a medicinal plant widely used in traditional Asian medicine. Andrographolide (10 mg/kg, ip, 4 weeks) improved CCH-induced cognitive deficits and hippocampal apoptosis, inhibited astrocyte activation and decreased the expression of TNF-α, IL-1β caspase-3 in the hippocampus, while alleviated 2VO-induced decreases in the expression of BDNF and TrkB (Wang et al., 2019). Andrographolide could reverse 2VO-induced activation of p-PTEN, while attenuated 2VO-decreased p-Akt. Thus, both BDNF/TrkB and PTEN/AKT signaling pathways were likely involved in neuroprotective effects of Andrographolide (Wang et al., 2020).

#### 4.6.2 Gypenoside (GP)

Gypenoside (GP) is a triterpenoid saponin extracted from *Gyrostemma pentaphyllum* (Thunnb.) Makino and exhibited multiple pharmacological effects. GP (400 mg/kg, po for 33 days) improved cognitive deficits in 2VO rats. Klüver–Barrera staining showed GP protection against CCH-induced axonal damage, and immunochemical stain for 4-HNE, 8-OHdG, and GAFP showed GP decreased oxidative stress and astroglia activation. WB showed that GP increased SOD and decreased MDA, demonstrating the therapeutic potential for GP to treat CCH (Zhang et al., 2011).

#### 4.6.3 Ginsenoside Rd

Ginsenoside Rd is a bioactive saponin compound primarily extracted from the roots of *Panax ginseng* (*Asian ginseng*) and is widely used in traditional medicine for their neuroprotective and anti-inflammatory properties. Ginsenoside Rd (10 and 30 mg/kg, ip for 21 days) alleviated CCH-induced learning and memory impairments in BCAS mice, and this improvement was linked to enhanced neuronal survival and increased BDNF expression in the hippocampus and front cortex, with increased p300/CBP and decreased expression of HDAC2. The implicated epigenetic regulation was further investigated in primary neuronal cell culture subjected to ODG/R, suggesting the epigenetics could account for BDNF increase to achieve neuroprotection (Wan et al., 2017).

#### 4.6.4 Pseudoginsenoside-F11 (PF11)

Pseudoginsenoside-F11 (PF11) is a triterpenoid saponin unique from *Panax quinquefolium* L., and has been shown to produce many neuroprotective effects. PF11 (6, 12, 24 mg/kg, po for 4 weeks) ameliorated gradual 2VO-induced cognitive impairment. PF11 alleviated white matter injury by improving the condition of the myelin sheath and axons and reducing their swelling. PF11 also prevented mature oligodendrocyte death and inhibited the activation of microglia and astrocytes. 2VO-increased p-mTOR, p-ULK1 and p-P70S6K were attenuated by PF11, thereby reducing the aberrant accumulation of autophagy substrates and increasing the level of autophagosomes in the white matter (Wang H. et al., 2024).

### 4.7 The neuroprotective effects of other compounds

#### 4.7.1 Ligustilide

Ligustilide is a dihydrophthalide compound primarily extracted from the roots of *Angelica sinensis* and *Ligusticum sinense* ‘chuanxiong’, both of which are widely used in TCM for their cardiovascular and neuroprotective properties. Ligustilide (80 mg/kg, po, 7 days) demonstrated cognitive deficit improvements in 2VO rats. Neuron loss was ameliorated as evidenced by Nissl staining and NeuN staining; Dendritic integrity was maintained as evidenced by MAP2 staining; while the staining for caspase-3, and GFAP was attenuated, indicating its anti-apoptosis and astroglia inhibition properties (Feng et al., 2012). In a recent study, Ligustilide (20, 40 mg/kg, po, 28 days) improved cognitive deficit in 2VO rats, increased Nissl positive cells. Ligustilide showed effects against ER stress and oxidative stress, The antioxidant SOD, CAT and GSH-Px were increased, while apoptosis protein Bax decreased. Ligustilide reversed 2VO-decreased SIRT1 and PDI, while suppressed 2VO-increased CHOP, XBP1, p-IRE1α. The downregulation of the IRE1α/XBP1 pathway by activating SIRT1 were further verified in PC12 cells under OGD, and these effects were partially blocked by SIRT1 inhibitor EX-527, indicating the critical role of SIRT1 in the protection (Peng et al., 2022).

#### 4.7.2 Imperatorin

Imperatorin is an active ingredient extracted from *Aangelica dahurica* (Baizhi) and other TCM herbs, and has many beneficial effects including neuroprotection. Imperatorin (2.5, 5, and 10 mg/kg, ip for 12 weeks) improved 2VO-induced cognitive impairments in MWM, alleviated hippocampal CA1 neuron loss. Mechanistically, Imperatorin inhibited apoptosis by upregulating the expression of Bcl-2, and downregulating Bax and caspase-3. Imperatorin improved synaptic ultrastructure, increased synaptic active zone length, PSD thickness, and the expression of PSD-95, suggesting the maintenance of synapse function could contribute to CCH treatment (Huang et al., 2021).

The TCM pure compounds against 2VO/BCAS CCH models were categorized similarly as TCM monomers against acute MCAO ischemia models (Xie et al., 2021). Some pure compounds have more publications against 2VO CCH models, but only representative ones were selected (i.e., resveratrol), some had already included in citated reference, and duplication is avoided, such as Curcumin against 2VO rat model (He et al., 2023). TCM pure compounds research greatly advance the pharmacological basis of TCM in alleviating CCH.

## 5 Discussion

### 5.1 The CCH model is different from acute MCAO model

This manuscript reviewed 50 publications on the protective effects of 11 TCM formulae, 8 herb extracts, and 21 pure compounds on CCH using 2VO, BCAS, and UCCAO rodent models. In general, 2VO could produce 10% mortality, which could be as high as 28% (Feng et al., 2012). Thus, gradual 2VO (ligation of the other side 1 weeks later, 6 publications) and UCCAO (1 publication) were used to avoid acute CBF reduction and acute neuroinflammation to more closely mimic cognitive deficits after white matter damage (Tuo et al., 2021; Kimura et al., 2025). BCAS is primarily applied to mice (7 publications) but also applied to rats (Zhilan et al., 2025). In BCAS/Mice model, the 0.18 mm diameter is frequently chosen to reduce the mortality with 0.16 mm and increase the severity with 0.22 mm microcoils to better mimic stenosis patients (Tuo et al., 2021). In these CCH models, compensatory blood supply occurs *via* the vertebrobasilar system over weeks to months, together with vascular remodeling, that helped to restore cerebral blood flow to pre-occlusion levels, preventing sustained long-term cerebral ischemia in sharp comparison to acute MCAO model that produced focal cerebral infarction within hours with high mortality rate (30%–60%) (Tuo et al., 2021; Kimura et al., 2025). Therefore, the mechanism of TCM protection using CCH and MCAO models shared some common but had some different mechanisms (Zhang et al., 2020; Fadoul et al., 2024). As a results, CCH treatment usually lasted for 4 weeks and even months after CCH modeling, compared to a few days of pretreatment and a few hours after MCAO modeling. Thus, acute ischemic stroke induced by MCAO over a short period does not fully align with the pathological progression of clinical patients (Kimura et al., 2025; Zhilan et al., 2025).

### 5.2 Cognitive improvement is the main parameter in CCH models

The improvement of cognitive impairments was the first line of evidence of protection in CCH models. Among 50 publications, Morris water maze (MWM) is the mostly performed test (88%), approximate 15% studies also used Novel object recognition, Open field and Y-maze tests, and 8% used passive avoidance and step-down tests to evaluate cognitive functions. In comparison, neurological scores is mainly used to evaluate the behavior abnormalities in MCAO model of acute cerebral ischemic stroke, and the outcomes of MWM in MCAO vary considerably and the conclusions are not always consistent (DeVries et al., 2001).

### 5.3 Histopathology of CCH vs. MCAO

HE and Nissl staining were most widely used to evaluate neuron damage and loss (88%) and TUNEL for apoptosis (18%). Transmission electron microscope was used to assess mitochondria, synapse, and white matter lesions from CCH (Niu et al., 2020b; Huang et al., 2021; Sun et al., 2021; Zhang et al., 2023; Wang H. et al., 2024; Wu et al., 2025). White matter includes nerve fibers (axons) covered by a fatty tissue called myelin. Reduced blood flow to the white matter can cause white matter injury responsible for cognitive deficits. Immunofluorescence and immunohistochemistry (65%) were widely used to locate selected cell-death molecules together with WB and qRT-PCR (Niu et al., 2020a; Yan et al., 2022; Pang et al., 2024; Wang H. et al., 2024; Xiao et al., 2024). Although these lesions were also observed in the MCAO brain, the TTC staining of infarction size is the golden histopathology for acute cerebral ischemic stroke.

### 5.4 Reduced CBF is more evident in MCAO than in CCH models

Reduced cerebral blood flow (CBF) is more evident in acute MCAO model than in chronic CCH models. However, CCH could activate a molecular and cellular cascade leading to breakdown of the blood-brain-barrier (BBB), that could initiate and contribute to a vicious cycle, causing neurodegeneration (Rajeev et al., 2022). Among 8 studies with CBF, three were used to verify the CCH model only (Jeong et al., 2020; Wang H. et al., 2024; Ji et al., 2025), Buqi Huoxue Tongnao (Gao et al., 2024) Xinshubao tablet (Xiao et al., 2024), and GJ-4 (Pang et al., 2024) could improve CBF in CCH models. However, Resveratrol (Fagerli et al., 2024) and Honokiol (Zhang et al., 2023) did not improve CBF. Thus, the improvement of CBF is ideal, but other mechanisms might also be responsible for the protective effects of TCM on CCH, and the maintenance of BBB is an important mechanism of TCM protection (Zhilan et al., 2025).

### 5.5 Anti-oxidative stress as one of the major protection mechanisms

Oxidative stress from endoplasmic reticulum (ER) stress and mitochondrial dysfunction is one important pathological features of CCH and MCAO (Rajeev et al., 2023; Kimura et al., 2025). Taohong Siwu decoction reduced GRP78 (Bip), p-eIF2α, p-IRE1α and ATF6 in 2VO rats (Fan et al., 2024), Ligustilide reduced Bip, p-IRE1α, XBP1, and CHOP in 2VO rats, probably mediated through upregulating SIRT1, as the SIRT1 inhibitor attenuated such effects (Peng et al., 2022).

One of the mechanisms of TCM against CCH is to improve mitochondrial dysfunction (Wang Y. et al., 2023). Approximate 10% manuscripts focused on mitochondria as a mechanism of protection, including ultrastructure, mitochondrial membrane potential, and mitochondrial proteins for ROS production, mitophagy, and cell death. TCM formulae appeared to be more effective in improving CCH-induced mitochondrial dysfunction. In addition to a review on 9 TCM formulae against CCH (Wang Y. et al., 2023), recent publications showed that Bushen-Yizhi regulated mitophagy with the improvement of mitochondrial membrane activity and lysosomal proteins (Xiao et al., 2023), Xinshubao tablet improved BCAS-induced ultrastructural alterations on mitochondria and synapses and reduced white matter injury (Xiao et al., 2024), and ShenmaYizhi Decoction improved mitochondrial ultrastructure and cognitive deficits through the AMPK/UCP2 signaling (Sun et al., 2021), indicating the better therapeutic efficacy of TCM formulae over monomers.

### 5.6 Anti-inflammation as one of the major protection mechanisms

Neuroinflammation contributes to main pathologies in both MCAO and CCH models and Toll-like receptor 4 (TLR4) is the key receptor responsible for immune and inflammatory reactions (Rajeev et al., 2023; Yawoot et al., 2025), and inhibition of neuroinflammation has been considered the one of major mechanisms of TCM protection in every studies.

Microglia activation is a normal physiological response to stimuli, however, microglia over-activation could produce excess ROS, which in turn triggers neuroinflammation for many neurodegenerative diseases including ischemia stroke and is a major mechanism of TCM protection against CCH (Zhang et al., 2024). The inhibition of microglia (including astroglia), reduction of ROS and inflammatory mediators account for 64% (32/50) of the manuscript reviewed, and applies for TCM formulae, extracts and pure compounds.

With anti-neuroinflammation *via* TCM, CCH-induced injury to synapses and myelin could be attenuated by maintaining the ultrastructure (Niu et al., 2020a; Huang et al., 2021; Xiao et al., 2024; Wu et al., 2025), and by modulating the related proteins such as PSD93, PSD95, MAP2, *etc.* (Nie et al., 2016; Huang et al., 2021; Wu et al., 2025). Thus, anti-neuroinflammation by TCM could help to maintain synaptic plasticity and myelin integrity in CCH models.

With anti-neuroinflammation by TCM, the abnormal accumulation of Aβ and phosphorylated Tau were reduced following treatment with Icariin (Li et al., 2015), Icariside II (Yin et al., 2018), and Gastrodin (Wu et al., 2023). Reduced Aβ and phosphorylated Tau accumulation could further play roles in improving cognitive deficits in CCH but also in AD.

### 5.7 Promote neuro-regeneration as one of the major protection mechanisms

TCM formulae and extracts have been demonstrated to promote tissue repair as exampled in myocardial ischemic infarction (Wang Y. et al., 2022), which emerges as the new direction to explore the mechanism of TCM protection against CCH. Indeed, Xinshubao tablet could rescue hippocampal neurogenesis dysfunction in BCAS mice (Xiao et al., 2024), Icariside II could improve neuron axon regeneration in CCH rats (Liu et al., 2019), Salidroside could promote angiogenesis through the Notch signaling pathway (Zhilan et al., 2025), while Modified Dioscorea pills promoted angiogenesis and microcirculation remodeling through the Ang/Tie signaling (Kuang et al., 2023). Pseudoginsenoside-F11 could increase mature oligodendrocytes in the corpus callosum (Wang H. et al., 2024). Thus, the promotion of neuro-regeneration could be a novel mechanism of TCM protection against CCH.

### 5.8 Advanced technologies to elucidate TCM protection mechanism

The advanced biotechnology provides opportunity of using Omics to profile/screen the potential targets of TCM protection against CCH. RNA-Seq was applied to study the protective effects of Chuanzhitongluo capsule (Wang Z. et al., 2024) and Xinshubao tablet (Xiao et al., 2024) in BCAS mice to reveal novel molecular events. Metabolomics were performed to profile CCH-induced abnormal brain energy metabolism (Wu et al., 2022; Zhang et al., 2022; Wu et al., 2023) and lipid metabolism impairment (Pang et al., 2024) and the protective effects of TCM. 16S rRNA-Seq was performed to assess whether the protective effects of Shunaoxin dropping pill against gradual 2VO rats was mediated through the microbiota-gut-brain axis (Bo et al., 2023). In addition, network pharmacology of Buqi Huoxue Tong nao against 2VO rats was performed to seek the potential signaling pathways of protection (Gao et al., 2024). Omics approaches could add our understanding of multi-target feature of TCM in the treatment of CCH.

To confirm and verify *in vivo* findings, a number of *in vitro* models were utilized together with CCH animal models. The most used *in vitro* model was OGD/R in primary neuron cultures (Wan et al., 2017; Wu et al., 2025), in microglial BV2 cells (Gao et al., 2024) and in PC12 cells (Peng et al., 2022; Xiao et al., 2023). The H_2_O_2_-treated hippocampal HT-22 cells (Wu et al., 2023) and OLN-93 cells (Pang et al., 2024) were also used. These *in vitro* studies affirmed *in vivo* findings and greatly advanced our understanding of molecular events responsible for TCM protection against CCH.

Neurotransmitter modulation could be another mechanism of TCM protection. For example, there are several manuscripts targeting cholinergic pathway as a protective mechanism against cognitive impairment, the increase in ChAT+ cells (Xu et al., 2009; Kim et al., 2016; Fagerli et al., 2024; Wang Z. et al., 2024) could improve cognitive deficits and modulate neuroinflammation. Anisodine hydrobromide, a brain cholinergic receptor modulator, could affect cholinergic system and neurotransmitter imbalance to exert protective effects in 2VO rats (Chen et al., 2017).

### 5.9 Signaling pathways

Under conditions of CCH, the protective effects of TCM could be derived from the synergistic modulation of core signaling pathways such as Nrf2/ARE, NF-κB, and BDNF/CREB by its multiple bioactive components. These pathways engage in extensive crosstalk: activation of Nrf2 not only enhances antioxidant defenses but also indirectly suppresses NF-κB-mediated inflammatory signaling, thereby disrupting the vicious cycle between oxidative stress and neuroinflammation (Fadoul et al., 2024). Meanwhile, the improved microenvironment facilitates the release of BDNF, which activates CREB-dependent neuroplasticity and regenerative processes. Enhanced neurotrophic signaling, in turn, further suppresses neuroinflammation and augments antioxidant capacity. Ultimately, through multi-pathway interactions, TCMs construct a coordinated regulatory network that collectively mitigate neuronal injury and promotes functional recovery.

The MAPK signaling pathways were frequently involved in TCM protection against CCH, such as *Ginkgo biloba* extracts (Kim et al., 2016), *Fructus mume* extracts (Lee et al., 2015), and *Scutellaria baicalensis* extracts (Hwang et al., 2011). The modulation of BDNF/TrkB pathways was implicated in the protective effects of Icariside II (Yin et al., 2018), Andrographolide (Wang et al., 2019), Ginsenoside Rd (Wan et al., 2017) and Epimedium flavonoids (Niu et al., 2020b). The modulation of the SIRT1/IRE1α/XBP1/CHOP pathway was implicated in Ligustilide protection in 2VO rats (Peng et al., 2022). The regulation of Nrf2/ARE antioxidant pathway was implicated in GJ-4 protection in BCAS mice (Pang et al., 2024). Other pathways such as PTEN and mTOR/Akt pathways as well as the epigenetic regulations were also investigated as the mechanism of protection.

## 6 Limitations and future perspectives

Upon a thorough search, we found that the clinical efficacy and safety reports using TCM in the treatment of CCH is limited in the literature despite the availability of these formulae in TCM drug stores/in hospital preparations and in clinical practices. Nonetheless, the preclinical findings could provide strong pharmacological basis and rationale for future clinical trials to evaluate the efficacy and safety of TCM formulae in patients with CCH or vascular cognitive impairment.

This article has reviewed 21 pure compounds extracted from TCM. However, these pure compounds are widely existed in the plant-kingdom, not unique to a certain herb originally extracted. These pure compounds belong to polyphenols, flavonoids, alkaloids, terpenoids, saponins, iridoid glycosides, glucosides, and other categories (Xie et al., 2021; Zheng et al., 2023; Shu et al., 2025). These components act together in designed recipes to play integrated roles, rather than exclude each other, to target multiple pathways. The strategy of “combination drug therapy” to enhance efficacy and reduce toxicity could be future perspectives.

## 7 Conclusion

**FIGURE 3:**
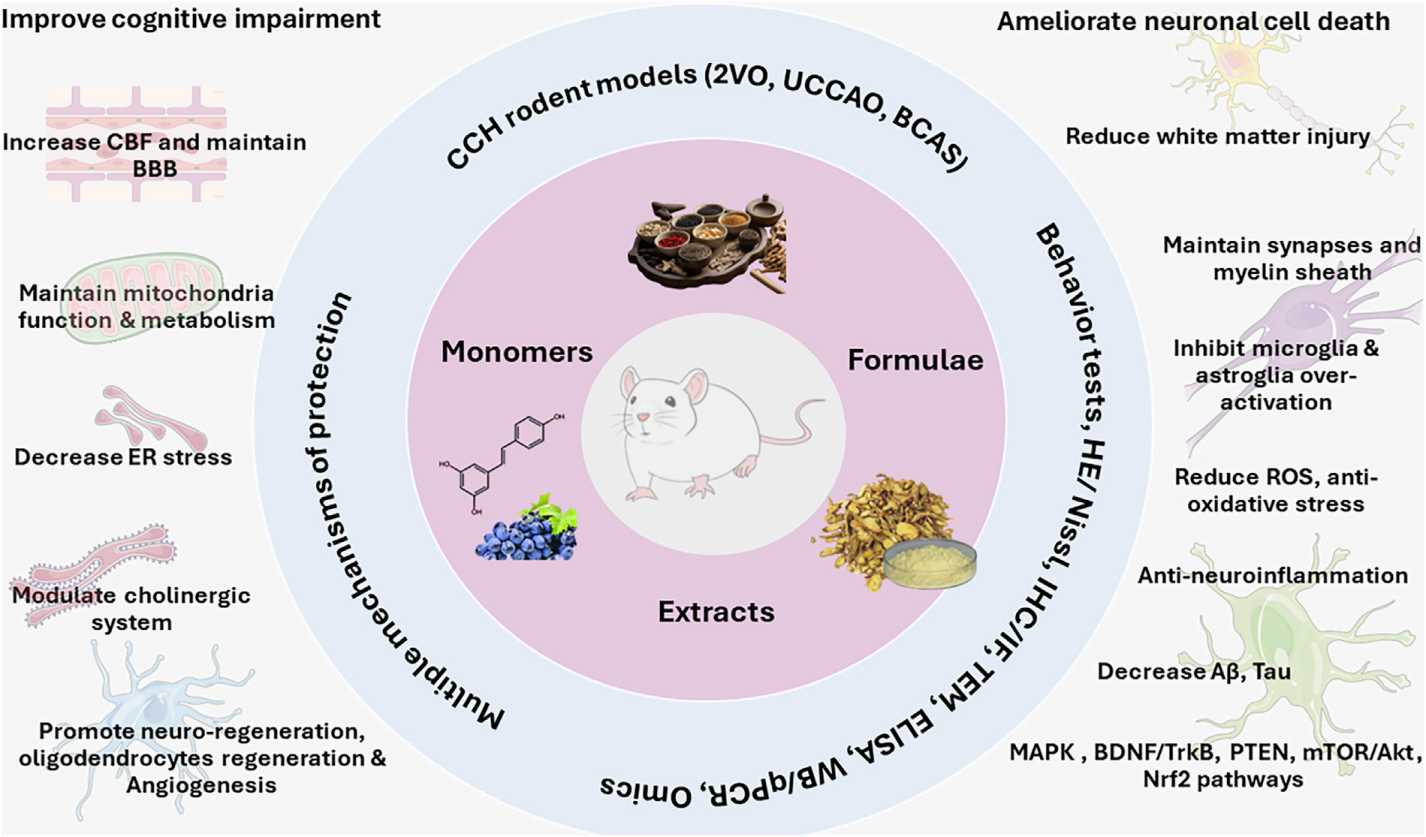
Multi-component and multi-target feature for TCM formulae, extracts, and pure compounds in the protection against chronic cerebral hypoperfusion. It should be noted that these protection mechanisms and signaling pathways are not mutually exclusive, rather, they function together in an integrated manner to protect CCH.

TCM demonstrated the clear therapeutic efficacy against CCH models, either from TCM formulae, herb extracts, or from pure compounds extracted from TCM. Many advanced techniques were used in ethnopharmacology investigations. It is clear that the protective effects of TCM against CCH are mediated through multi-component and multi-target mechanisms as summarized in Figure 3.
